# The optimized acupuncture treatment for neck pain caused by cervical spondylosis: a study protocol of a multicentre randomized controlled trial

**DOI:** 10.1186/1745-6215-13-107

**Published:** 2012-07-09

**Authors:** Zhao-Hui Liang, Zhong Di, Shuo Jiang, Shu-Jun Xu, Xiao-Ping Zhu, Wen-Bin Fu, Ai-Ping Lu

**Affiliations:** 1 Research Team of Acupuncture Effect and Mechanism, Guangdong Provincial Academy of Chinese Medical Sciences, Guangzhou 510120, China; 2 Guangdong Provincial Hospital of Chinese Medicine, Number 111, Dade Road, Guangzhou 510120, China

**Keywords:** Acupuncture, Cervical spondylosis, Neck pain

## Abstract

**Background:**

Neck pain is one of the chief symptoms of cervical spondylosis (CS). Acupuncture is a well-accepted and widely used complementary therapy for the management of neck pain caused by CS. In this paper, we present a randomized controlled trial protocol evaluating the use of acupuncture for CS neck pain, comparing the effects of the optimized acupuncture therapy in real practice compared with sham and shallow acupuncture.

**Methods/Design:**

This trial uses a multicentre, parallel-group, randomized, sham acupuncture and shallow acupuncture, controlled single-blind design. Nine hospitals are involved as trial centres. 945 patients who meet inclusion criteria are randomly assigned to receive optimized acupuncture therapy, sham acupuncture or shallow acupuncture by a computerized central randomization system. The interventions past for 4 weeks with eight to ten treatments in total. The group allocations and interventions are concealed to patients and statisticians. The Northwick Park Neck Pain Questionnaire (NPQ) is used as the primary outcome measure, and the McGill Pain Questionnaire (MPQ) and The Short Form (36) Health Survey (SF-36) are applied as secondary outcome measures. The evaluation is performed at baseline, at the end of the intervention, and at the end of the first month and the third month during follow-up. The statistical analyses will include baseline data comparison and repeated measures of analysis of variance (ANOVA) for primary and secondary outcomes of group and time differences. Adverse events (AEs) will be reported if they occur.

**Discussion:**

This trial is a multicentre randomized control trial (RCT) on the efficacy of acupuncture for CS neck pain and has a large sample size and central randomization in China. It will strictly follow the CONSORT statement and STRICTA extension guideline to report high-quality study results. By setting the control groups as sham and shallow acupuncture, this study attempts to reveal the effects of real acupuncture versus placebo or non-classic acupuncture treatment and evaluate whether classic Chinese medical acupuncture is effective on CS neck pain. This study will provide evidence for the effects of acupuncture on CS neck pain.

**Trial Registration:**

Chinese Clinical Trial Registry: ChiCTR-TRC-00000184.

## Background

Cervical spondylosis (CS) is caused by unspecified degenerative changes of the muscles, tendons, joints, and bones of the neck and shoulder [1]. The main symptom of CS is pain and stiffness in the neck, sometimes accompanied by numbness and radicular pain to the arms and fingers. The overall prevalence of neck pain has been reported [2] as ranging from 0.4% to 86.8% (mean 23.1%). In a high-risk population (office and computer workers), the 1-year incidence of neck pain ranged from 10.4% to 21.3% [2], and most of these cases are empirically determined to be caused by CS (hereafter referred to as CS neck pain).

The management goals for CS neck pain are to diminish pain intensity, facilitate physical neck movement, and restore daily function. Therapies for the disorder include both individual (administration of non-steroidal anti-inflammatory drugs), muscle relaxants, and analgesics [3]), and public (establishment of healthcare policies and the delivery of healthcare advice, physical training and stress management techniques [4]) treatment approaches. However, little evidence is available to support the effectiveness of care at either the public or individual healthcare level [3,4].

Complementary therapies such as acupuncture, massage, neck exercises, mechanical traction, and electrotherapy are well accepted as alternative treatments for CS neck pain. Acupuncture has been extensively used for the management of CS neck pain, and a number of clinical trials have been conducted to test its efficacy. However, the evidence remains moderate, because of defects in study design and in evaluation methods used in current systematic reviews [5].

One problem is that many new acupuncture methods have been introduced in these studies. For example, trigger-point acupuncture was reported to be effective for chronic neck pain in elderly patients [6]. Another study reported that tender-point acupuncture had short-term effects on neck and shoulder pain and stiffness [7]. Traditional acupuncture therapy is based on the classical knowledge of Chinese medicine, and acupuncture point selection should follow those classical principles and specific locations. Thus, the aforementioned studies provide evidence only for the efficacy of needle insertion, not for the efficacy of acupuncture *per se*.

Problems in the design of some studies have also been identified. Some trials had a small sample size without the correct calculation of the sample size required to give meaningful statisics [6-8], whereas others lacked a meaningful control group [9] or blinding techniques to prevent subjective bias [10,11]. Therefore, a well-designed, multicentre RCT with sound methods for calculating sample size and establishing a meaningful control group is needed to evaluate the effects of classic Chinese acupuncture for the treatment of CS neck pain.

The current study was designed as a randomized, controlled, single-blind study, covering nine centres and comparing three groups (one treatment group and two control groups).. The sample size was calculated based on the data from a 178-case pilot study [1] and referred to the large-scale pragmatic trial design recommendation proposed by Salter *et al*. [12]. The objective of the trial was to evaluate the effects of optimized acupuncture therapy (OAT) for CS neck pain.

The trial protocol is reported here in accordance with CONSORT 2010 statement [13], and the intervention acupuncture details are described in accordance with the STRICTA 2010 extension [14,15].

## Methods/Design

### Ethics review and informed consent

The study protocol was reviewed and approved by the Ethics Committee of Guangdong Provincial Hospital of Chinese Medicine (number 2008GL-10),and registered in the Chinese Clinical Trial Registry (number ChiCTR-TRC-00000184), which is a primary registry in the WHO registry network (http://www.chictr.org/). All patients are required to provide informed consent.

### Trial status

Patients are currently being recruited into the study, and the follow-up is being conducted.

### Trial design

The study was designed as a randomized, parallel-group, single-blind trial to evaluate the effects of OAT for CS neck pain. The following nine hospitals located in different regions of China were chosen for involvement in the trial: Guangdong Provincial Hospital of Chinese Medicine, the First Affiliated Hospital of Hunan University of Chinese Medicine, the Secondary Affiliated Hospital of Hunan University of Chinese Medicine, Xinjiang Medical University Affiliated Hospital of Traditional Chinese Medicine, the Third Hospital of Shanxi Institute of Traditional Chinese Medicine, the Second Affiliated Hospital of Guiyang Institute of Traditional Chinese Medicine, the People’s Hospital of Hainan Province, the Second People’s Hospital of Zhaoqing City, and the People’s Hospital of Huizhou City.

### Study procedure and patient recruitment

**Figure 1:**
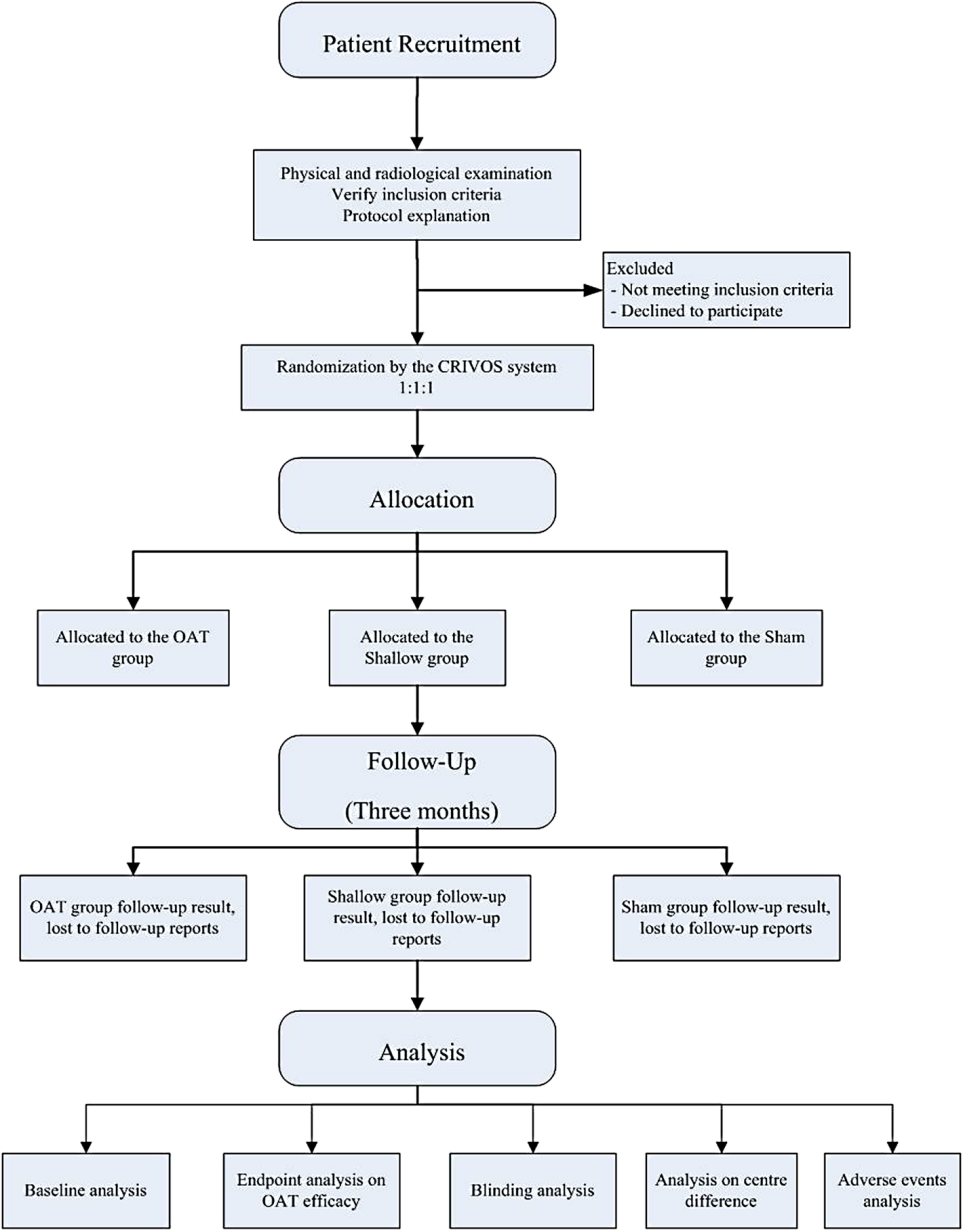
Patient recruitment.

The study procedure is illustrated in the flow chart in Figure 1.

The researchers will distribute trial information leaflets to the outpatient clinics of the hospitals, local communities, and university campuses to recruit potential patients whose major symptoms are neck pain and stiffness. The leaflets contain a brief questionnaire for screening patients with CS and provide contact information for the researcher. The patients are given a physical examination and radiography assessment to confirm the diagnosis of CS, and the protocol details are explained to them. Patients are included only if they meet the inclusion criteria and sign an informed consent document.

#### Inclusion and exclusion criteria

The inclusion criteria for the trial are:

· Age 18 to 60 years (both males and female patients are included).

· A confirmed diagnosis of CS in accordance with the diagnostic criteria published by Chinese Medical Association and referring to the *International Classification of Diseases*, 10th edition (ICD-10) [16] codes: M47.2 (other spondylosis with radiculopathy), M47.8 (other spondylosis → cervical spondylosis), or M47.0 + G99.2* (anterior spinal and vertebral artery compression syndromes).

· Diagnosis of CS supported by a cervical radiographic examination, such as anteroposterior and lateral X-rays, or magnetic resonance imaging/computed tomography scans showing cervical spine degeneration or cervical disc herniation.

· The main symptom of neck pain.

· Episodes of neck pain episodes lasting at least 0.5 hours, and occurring at a frequency of at least once per month for the 3 months prior to study entry.

· Pain intensity of more than 3 points as measured on a visual analog scale (VAS) at the time of recruitment.

· No acupuncture therapy within the 3 months prior to study entry.

The exclusion criteria are:

· Pregnancy or lactation.

· Confirmed diagnosis of spondylogenic compression of the spinal cord referring to the ICD-10 [16] code M47.1 + G99.2*.

· Complications of severe systematic diseases such as diabetes mellitus, cardiocerebrovascular disorders or tumors

· Considered not suitable to participate the trial by the researchers.

· History of neck trauma, cervical fracture or cervical surgery, neurologic impairment (for example, myasthenia or abnormal spinal nerve reflex), or congenital spinal abnormality, or a diagnosis of systemic disease of the bones or joints.

· Duration of neck pain and/or neck stiffness episodes of less than 0.5 hours or history of episodes have occurred over less than < 3 months.

· Rejection or fear of acupuncture therapy.

· Use of any type of acupuncture within the 3 months prior to study entry.

· Use of any other treatments (drug or non-drug).

### Data collection

The demographic, baseline characteristics, and clinical outcome data will be collected in the clinics where the patients are treated. The questionnaires to assess treatment effects and physical conditions are to be completed by the patients after the treatment is completed. During the follow-up period, the participants are requested to return to the clinics to provide follow-up information and will be given subsidies to cover transportation and lost income based on the local minimum wage.

### Treatment regimen

Patients are required to receive eight to ten treatment sessions, at a frequency of two to three times a week. The intervention should be completed in 4 weeks.

### Interventions

The treatment scheme originates from the classical principles of traditional Chinese medicine and has been practiced in Guangdong Provincial Hospital of Chinese Medicine for some time. The final scheme was discussed and revised with the advice from a national expert committee of acupuncturists organized by the State Administration of Traditional Chinese Medicine (SATCM) of China. We hypothesized that OAT would have a specific effect on CS neck pain and be superior to both placebo and non-specific effects, therefore, two control groups were designed, in addition to the treatment group, as detailed below.

#### The optimized acupuncture therapy group

The treatment scheme for the OAT group consists of traditional acupuncture therapy, followed by intradermal needle therapy (INT). Nine acupuncture points were selected for the OAT group based on the advice and consensus of the national expert committee of acupuncturists detailed above. The name/code and location of the acupuncture points followed the WHO standards where possible [17-19], and are: Jianzhongshu (SI15, bilateral); Dazhui (GV14); Zhongzhu (TE3, bilateral); and Huatuojiaji (extra point; four points in two pairs, bilateral). The Huatuojiaji points are traditional Chinese acupuncture points, but are not listed in the WHO standard. To locate these four points, the acupuncturist needs to identify the cervical positive reaction planes by palpating the area 12.5 mm away from the patient’s cervical vertebra. If any tender points are found, the corresponding cervical vertebra level is considered the cervical positive reaction plane. The four Huatuojiaji points are located at the top ( upper Huatuojiaji) and bottom (lower Huatuojiaji) of th cervical positive reaction plane(s) bilaterally and 12.5 mm horizontally away from the corresponding cervical vertebra. If only one cervical positive reaction plane is identified, the practitioner should select a pair of the Huatuojiaji points on the cervical positive reaction plane and a second pair on the cervical vertebra next to the first pair.

The doctors performing all therapies have at least 5 years of acupuncture experience. Disposable stainless steel needles (0.3 mm × 40 mm; Suzhou Tianxie Acupuncture Instruments Co. Ltd, Su Zhou City, China) are used. The needles are inserted into the muscular tissue of the acupuncture points (to a depth of 20 mm) using the tube-guide method. The inserted needles are manually manipulated until the patient feels numbness or other acupuncture sensation (known as ‘de qi’), and the needles will be retained in the points for 20 minutes.

Following this tradition acupuncture therapyt, patients are treated by INT. There are two point selection schemes used alternatively: 1) the upper Huatuojiaji (bilateral) and Dazhui (GV14) and 2) the lower Huatuojiaji (bilateral) and Jianzhongshu (SI15; bilateral). Disposable stainless steel intradermal needles (0.22 mm × 5 mm; Suzhou Tianxie Acupuncture Instruments Co. Ltd.) are inserted into the skin to the subcutaneous tissue at an angle of 15 degrees to the point surface. The direction of needle insertion in Dazhui (GV14) is parallel to the spine, and in Huatuojiaji and Jianzhongshu (SI15), it is vertical to the spine. The inserted needles are fixed with medical tape, with their hilts remaining outside the skin, and will not be removed until the next treatment session.

#### The sham acupuncture group

Skin-penetrating shallow needles on sham points are used in the sham group. The location of the sham points is defined as 25 mm lateral to the standard location used in the OAT group: the sham point of Dazhui (GV14) is 25 mm vertically below the standard GV 14, the sham points of Jianzhongshu (SI15) and Huatuojiaji are 25 mm lateral to the standard SI15 and the corresponding points of Huatuojiaji, and the sham point of Zhongzhu (TE3) is 25 mm proximal to the standard Zhongzhu (TE3). The same type of disposable stainless-steel needles is used in the sham group as in the OAT group, therefore, the intervention in the sham group cannot be differentiated by needle appearance. The needles (0.22 mm × 40 mm) are inserted into the skin to a depth of 3 mm and remain at the subcutaneous level for 20 minutes. The doctor uses the tube-guide method, and any manipulation for acupuncture sensation or de qi is prohibited.

After sham acupuncture, the patients will be treated by point pressing. The point selection scheme is the same as for the INT in the OAT group. To ensure blinding, Semen Vaccariae (the seeds of the cow cockle, *Vaccaria hispanica*) are used to press on each point to conceal from the patient whether INT is being administered; as the points are all on the back, the patient cannot see what is being pressed on their skin.

#### The shallow acupuncture group

For the shallow group, the point selectionp is the same as for the OAT group; however, the doctor is required to insert the needles vertically to the subcutaneous level at a depth of no more than 3 mm. Any sensation (de qi) or needle manipulation is prohibited in this group. After acupuncture, the patients will also treated by INT using the same method as in the OAT group.

### Randomization

Randomization is performed using a central randomization interactive voice operating system (CRIVOS) developed by the Design, Measurement and Evaluation in Clinical Research (DME) centre of Guangzhou University of Chinese Medicine. The random allocation sequence was generated by an independent statistician from the DME centre using SAS software (SAS Institute, Cary, NC, USA), which was uploaded to CRIVOS. The doctors and researchers from all centers can connect to the CRIVOS system by telephone at any time to acquire the allocation result when they are enrolling participants. To enroll a patient, the doctor first confirms the patient qualifies for inclusion, then the doctor telephones the CRIVOS system and follows the voice instruction given, and finally, the doctor records the allocation information. There are three numbers indicating the allocation result: 001 represents the OAT group, 002 represents the shallow group, and 003 represents the sham group.

### Blinding

Because the efficacy of acupuncture inevitably depends on the skill of the operating doctors, allocation concealment to the doctors is not feasible for trials on acupuncture. Consequently, we applied a single-blind design in which the patients are blinded to their allocation. The statisticians are blinded to all treatment allocations throughout the study.. The concealment will not be broken until the final data analysis report is completed.

During and intervention phrase, the participants are treated with tube-guided needles that have the same appearance as the treatment needles. Similarly, use of the seeds in the sham acupuncture group gives the patient the sensation of a needle being inserted. Both the genuine acupuncture points and the sham points are in similar locations on the patients’ neck, back, and hands, and the participants cannot observe the manipulation performed during the treatment, therefore, the blinding should be effective.The requirements for the treatment sessions (the duration and frequency) are the same. After the second session and at the end of the treatment, participants are required to complete a questionnaire to assess the blinding.

### Outcomes

Three well-recognized patient-reported outcome (PRO) tools are used to measure the effect outcomes. The Northwick Park Neck Pain Questionnaire (NPQ) is used as the primary outcome measure, and the McGill Pain Questionnaire (MPQ) and the Medical Outcomes Study Short Form 36 (SF-36) are used as secondary outcome measures.

#### Primary outcome

The NPQ [20] is composed of nine items that measure: 1) neck-pain intensity, 2) relationship between neck pain and sleeping, 3) sensation of pins and needles in the arms at night, 4) duration of symptoms, 5) ability to carry items, 6) read and watch TV, 7) ability to work/perform housework, 8) ability to perform social activities (for example, doing leisure activities, visit friend.); and 9) ability to drive. The NPQ produces an overall percentage score, with a higher score reflecting a more serious disease state.

#### Secondary outcomes

The MPQ is a classic tool proposed by Melzack [21] for assessing the intensity of non-specific pain, and a higher MPQ score reflects more serious pain. The SF-36 is a general tool to measure medical outcomes and quality of life (QOL) [22,23]. The SF-36 consists of assessing outcomes in eight domains to represent patient QOL in physical, mental and social dimensions. A higher score reflects a better QOL in the corresponding domain.

In this trial, analgesic use is not recommended. Howver, based on bioethical considerations, the researchers will be able to provide free ibuprofen sustained-release capsules (Fenbid; GSK-China, Shanghai, China); State Food and Drugs Administration (China) registration number H10900089.) to the included subjects who request analgesic. The dose is 0.3 g (one capsule) orally, twice daily (morning and evening). The subjects’ analgesic use will be recorded and any use of analgesic beyond this will be considered a protocol violation.

#### Outcome measuring procedure and data collection

The outcomes will be assessed before the first treatment (baseline measurement), immediately after the final treatment (short-term outcome of therapeutic effect), and at the end of the first and third months after the final treatment (long-term effective outcomes). The data will be by PRO, with the questionnaires being completed by the participants themselves with necessary instruction from the researchers. Medication use at baseline, during the intervention, and at the 3-month follow-up will be recorded.

### Safety evaluation

Any expected and unexpected adverse events (AEs) will be recorded during the treatment. The expected AEs include local bleeding at the needle insertion points, and local numbness, pain, and dizziness during treatment. If any AE occurs, the doctor will provide corresponding treatment to the patient in accordance with the study protocol. The researcher will complete the adverse events/reaction form in the case report form and report the AE to the primary investigator and ethics committee immediately, who will make a decision on whether the patient needs to withdraw from the trial.

### Sample size calculation

The size calculation is based on the mean and standard deviation (SD) for NPQ from a pilot study [1]. The reported pilot study was a two-arm design with a treatment group and one placebo group, whereas this trial protocol is a three-arm design with one treatment group (OAT) and two control groups (sham group and shallow group). The mean ± SD of the treatment group reported in the pilot study (20.71 ± S 11.91) [1] were used as the expected values of the OAT group, and the mean ± SD of the placebo group (24.04 ± 11.83) were used as the expected values of the two control groups. The software Power Analysis and Sample Size (PASS; version 11; NCSS Statistical Software, Kaysville, UT, USA) was used to perform the sample size calculation.

The sample size was calculated with a significance level of 0.05 and power of 0.90. The result was a total required sample size of 732, with 244 for each group. With a maximum dropout tolerance of 15%, 32 patients per center are needed for each group, or 94 patietns overall per center. Therefore, 846 patients are needed for the trial, with 288 for each group across the nine centers.

### Statistical analyses

EpiData software (version 3.1 is used for data entry, and SPSS (version 16.0; SPSS Inc., Chicago, IL, USA) for statistical analyses. The analytic strategy includes the descriptive analysis of the demographic characteristics and the baseline data for the primary and secondary outcomes. The group differences are tested by analysis of variance (ANOVA) for continuous variables, the Kruskal-Wallis test for ranking variables, and the χ² test for categorical variables. The significance level is set at α = 0.05, two-sided.

There are four measuring points set to assess the efficacy of the OAT, as described above in the Outcomes section. Therefore, we will apply the two-factor repeated measures analysis of variance (ANOVA) model with the time of measure as the within-subjects factor and treatment as the between-subjects factor (significance level α = 0.05, two-sided). In addition, stratification analysis will be used to explore the potential effect difference between centres.

## Discussion

The objective of this trial is to evaluate the effect of the OAT scheme for neck pain in patients with CS. The first draft of the OAT treatment scheme was developed based on the practice and clinical experience of the Acupuncture Department of Guangdong Provincial Hospital of Chinese Medicine. The draft was then discussed by a special committee of Guangdong Association of Acupuncture and revised. The revised edition was submitted and reviewed by the national expert committee of acupuncturists organized by SATCM, and the final version of the OAT scheme was approved by the consensus of the committee.

The OAT scheme comprises two steps: the first step is classic acupuncture therapy at the nine standard points with de qi sensation for 20 minutes, and the the second step is the administration of INT in which the needles are fixed by medical tape. The treatment lasts for 4 weeks with eight to ten sessions at a frequency of two to three times per week. The overall effect of OAT is related to the duration and frequency of the treatment. The intradermal needles are an empirical method to maintain the pain-relieving effect of classic acupuncture, therefore, the treatment frequency of classic acupuncture can be reduced to twice per week. As a result, both the final outcome and patient compliance are improved.

For a high-quality RCT, use of the appropriate control is crucial. In this trial, two questions must be addressed. First, is the OAT treatment effective on CS neck pain? Second, does the OAT treatment have a superior effect to the control treatments in treating CS neck pain? The sham and shallow acupuncture groups were designed as control groups in this study to answer these questions.

Sham acupuncture is considered to be a placebo treatment in acupuncture studies. In general, sham acupuncture can be divided into two categories: non-penetrating and penetrating sham acupuncture, which is determined by whether the needle penetrates the skin during the intervention. According to the literature, there are four types of non-penetrating sham acupuncture in use: 1) skin touch on standard points or non-points [24,25], 2) blunt telescoping sham needle [26,27], 3) transcutaneous electrical stimulation [28], and 4) sham laser acupuncture [29,30]. It is reported the non-penetrating sham acupuncture has been applied as a placebo in numerous trials; however, it was also found that 40% of the subjects in these trials could distinguish the sham acupuncture from the real treatment [31], breaking the blind concealment. Therefore, we considered that non-penetrating sham acupuncture would not be a useful placebo for this trial. However, two types of penetrating sham acupuncture are also available: 1) non-point deep puncturing needle insertion [32,33] and 2) non-point superficial needle insertion [34,35]. Although skin penetration is believed to cause some therapeutic effect such as diminished pain intensity, it is considered non-specific, and can be caused by other interventions. The greater the insertion depth and the more stimulation induced by the needle, the greater the effect. Consequently, we chose to usethe non-point superficial needle-insertion method for the trial. The needles are inserted into the sham points to a depth of 3 mm and are retained at the subcutaneous level. Because the needles are inserted on the patient’s back, the patients can sense the needle penetration but cannot distinguish the sham acupuncture from the real one. Therefore, we believe that this method can serve as an effective placebo intervention.

Considering the trial results, both pain intensity and physical function are subjective feelings, therefore, we will use the PRO and the validated scales and questionnaires to assess the clinical outcome. For the evaluation of CS neck pain, three aspects were included in the complete assessment: neck pain, physical function, and QOL. The NPQ will be used as the primary outcome to evaluate CS-related neck pain, radiculopathy. and impairment of daily functions, while for the secondary outcomes, the MPQ will measure the pain intensity in general settings, and the SF-36 will measure patient QOL changes caused by changes in physical and mental conditions.

Salter *et al *. [12] proposed a protocol for an RCT to evaluate the effect of acupuncture for neck pain, recommending 229 cases for each arm based on the assumption of 90% power to detect a five-point NPQ difference, a 5% significance level, and a 14% drop-out rate during follow-up. In our study, a total of 945 cases are required from 9 centres, therefore, there will be 315 cases for each arm, and at least 268 cases are required to complete the trial and follow-up. The report of the study results will follow the CONSORT 2010 guidelines [13], and the operation details for acupuncture will be reported in accordance with the 2010 CONSORT-STRICTA extension [14] guidelines. Thus, this study will be able to provide high-quality evidence for the efficacy of the OAT for CS neck pain.

In conclusion, the multi-centre RCT design of this trial will be able to assess and differentiate the effects of the OAT.

## Abbreviations

ChiCtr, Chinese Clinical Trial Register; CRIVOS, Central Randomisation Interactive Voice Operating System; DME, Design, Measurement and Evaluation in Clinical Research; MPQ, McGill Pain Questionnaire; NPQ, Northwick Park Neck Pain Questionnaire; OAT, Optimized acupuncture therapy; PRO, Patient-reported outcome; SATCM, State Administration of Traditional Chinese Medicine; SF-36, The Medical Outcomes Study Short Form 36-item Health Survey.

## Competing interests

The authors state that there are neither actual nor potential conflicts of interest, including any financial or personal relationships with other people or organisations since the work was submitted.

## Authors’ contributions

LZH drafted and revised the paper in accordance with the 2010 CONSORT & STRICTA guidelines, developed the statistical analysis strategy, smf submitted the protocol to ethics review. DZ participated the clinical study. JS was involved the study design and helped to draft the manuscript. XSJ and ZXP registered the protocol in the Chinese Cochrane Centre and developed the statistical plan. FWB organized the study, and LAP gave important advice on the study methodology. All authors read and approved the final manuscript.

## References

[B1] Liang Z Zhu X Yang X Fu W Lu A Assessment of a traditional acupuncture therapy for chronic neck pain: a pilot randomised controlled study Complement Ther Med 2011 19 Suppl 1 S26 S32 21195292 10.1016/j.ctim.2010.11.005

[B2] Hoy DG Protani M De R Buchbinder R The epidemiology of neck pain Best Pract Res Clin Rheumatol 2010 24 783 792 21665126 10.1016/j.berh.2011.01.019

[B3] Borenstein DG Chronic neck pain: how to approach treatment Curr Pain Headache Rep 2007 11 436 439 18173978 10.1007/s11916-007-0230-4

[B4] Cassidy JD Cote P Is it time for a population health approach to neck pain? J Manipulative Physiol Ther 2008 31 442 446 18722199 10.1016/j.jmpt.2008.06.008

[B5] Trinh K Graham N Gross A Goldsmith C Wang E Cameron I Kay T Acupuncture for neck disorders Spine (Phila Pa 1976) 2007 32 236 243 17224820 10.1097/01.brs.0000252100.61002.d4

[B6] Itoh K Katsumi Y Hirota S Kitakoji H Randomised trial of trigger point acupuncture compared with other acupuncture for treatment of chronic neck pain Complement Ther Med 2007 15 172 179 17709062 10.1016/j.ctim.2006.05.003

[B7] Nabeta T Kawakita K Relief of chronic neck and shoulder pain by manual acupuncture to tender points–a sham-controlled randomized trial Complement Ther Med 2002 10 217 222 12594972 10.1016/s0965-2299(02)00082-1

[B8] He D Veiersted KB Hostmark AT Medbo JI Effect of acupuncture treatment on chronic neck and shoulder pain in sedentary female workers: a 6-month and 3-year follow-up study Pain 2004 109 299 307 15157691 10.1016/j.pain.2004.01.018

[B9] White AR Ernst E A systematic review of randomized controlled trials of acupuncture for neck pain Rheumatology (Oxford) 1999 38 143 147 10342627 10.1093/rheumatology/38.2.143

[B10] White P Lewith G Prescott P Conway J Acupuncture versus placebo for the treatment of chronic mechanical neck pain: a randomized, controlled trial Ann Intern Med 2004 141 911 919 15611488 10.7326/0003-4819-141-12-200412210-00007

[B11] Giles LG Muller R Chronic spinal pain: a randomized clinical trial comparing medication, acupuncture, and spinal manipulation Spine (Phila Pa 1976) 2003 28 1490 1502 discussion 1502–1493 12865832 10.1097/00007632-200307150-00003

[B12] Salter GC Roman M Bland MJ MacPherson H Acupuncture for chronic neck pain: a pilot for a randomised controlled trial BMC Musculoskelet Disord 2006 7 99 17156464 10.1186/1471-2474-7-99 PMC1713236

[B13] Antes G The new CONSORT statement BMJ 2010 340 c1432 20332507 10.1136/bmj.c1432

[B14] MacPherson H Altman DG Hammerschlag R Youping L Taixiang W White A Moher D Revised STandards for Reporting Interventions in Clinical Trials of Acupuncture (STRICTA): extending the CONSORT statement J Altern Complement Med 2010 16 ST1 ST14 20954957 10.1089/acm.2010.1610

[B15] MacPherson H Jobst KA Improving the reporting of interventions in clinical trials of acupuncture: the updated and revised STRICTA J Altern Complement Med 2010 16 929 930 20839969 10.1089/acm.2010.0558

[B16] International Statistical Classification of Diseases and Related Health Problems 10th Revision Version for 2007 http://apps.who.int/classifications/apps/icd/icd10online/

[B17] WHO: A Proposed Standard International Acupuncture Nomenclature: Report of a WHO Scientific Group http://apps.who.int/medicinedocs/en/d/Jh2947e/4.3.html

[B18] Choi SH WHO traditional medicine strategy and activities. "Standardization with evidence-based approaches" J Acupunct Meridian Stud 2008 1 153 154 20633469 10.1016/S2005-2901(09)60037-6

[B19] WHO WHO Standard acupuncture point locations in the western pacific region 2008 World Health Organization, Geneva

[B20] Leak AM Cooper J Dyer S Williams KA Turner-Stokes L Frank AO The Northwick Park Neck Pain Questionnaire, devised to measure neck pain and disability Br J Rheumatol 1994 33 469 474 8173853 10.1093/rheumatology/33.5.469

[B21] Melzack R The McGill Pain Questionnaire: major properties and scoring methods Pain 1975 1 277 299 1235985 10.1016/0304-3959(75)90044-5

[B22] Ruta D Garratt A Abdalla M Buckingham K Russell I The SF 36 health survey questionnaire. A valid measure of health status BMJ 1993 307 448 449 8374477 10.1136/bmj.307.6901.448-b PMC1678408

[B23] Lam CL Tse EY Gandek B Fong DY The SF-36 summary scales were valid, reliable, and equivalent in a Chinese population J Clin Epidemiol 2005 58 815 822 16018917 10.1016/j.jclinepi.2004.12.008

[B24] White AR Resch KL Chan JC Norris CD Modi SK Patel JN Ernst E Acupuncture for episodic tension-type headache: a multicentre randomized controlled trial Cephalalgia 2000 20 632 637 11128820 10.1111/j.1468-2982.2000.00097.x

[B25] Streitberger K Kleinhenz J Introducing a placebo needle into acupuncture research Lancet 1998 352 364 365 9717924 10.1016/S0140-6736(97)10471-8

[B26] Park J White AR Ernst E New sham method in auricular acupuncture Arch Intern Med 2001 161 894 author reply 895 11268237 10.1001/archinte.161.6.894

[B27] Thorsteinsson G Stonnington HH Stillwell GK Elveback LR The placebo effect of transcutaneous electrical stimulation Pain 1978 5 31 41 353652 10.1016/0304-3959(78)90022-2

[B28] Irnich D Behrens N Gleditsch JM Stor W Schreiber MA Schops P Vickers AJ Beyer A Immediate effects of dry needling and acupuncture at distant points in chronic neck pain: results of a randomized, double-blind, sham-controlled crossover trial Pain 2002 99 83 89 12237186 10.1016/s0304-3959(02)00062-3

[B29] Konig A Radke S Molzen H Haase M Muller C Drexler D Natalis M Krauss M Behrens N Irnich D Randomised trial of acupuncture compared with conventional massage and "sham" laser acupuncture for treatment of chronic neck pain - range of motion analysis Z Orthop Ihre Grenzgeb 2003 141 395 400 12928995 10.1055/s-2003-41566

[B30] White P Lewith G Hopwood V Prescott P The placebo needle, is it a valid and convincing placebo for use in acupuncture trials? A randomised, single-blind, cross-over pilot trial Pain 2003 106 401 409 14659523 10.1016/j.pain.2003.08.013

[B31] Fink M Wolkenstein E Karst M Gehrke A Acupuncture in chronic epicondylitis: a randomized controlled trial Rheumatology (Oxford) 2002 41 205 209 11886971 10.1093/rheumatology/41.2.205

[B32] Shapira MY Berkman N Ben-David G Avital A Bardach E Breuer R Short-term acupuncture therapy is of no benefit in patients with moderate persistent asthma Chest 2002 121 1396 1400 12006419 10.1378/chest.121.5.1396

[B33] Zaslawski C Rogers C Garvey M Ryan D Yang CX Zhang SP Strategies to maintain the credibility of sham acupuncture used as a control treatment in clinical trials J Altern Complement Med 1997 3 257 266 9430329 10.1089/acm.1997.3.257

[B34] Goddard G Karibe H McNeill C Villafuerte E Acupuncture and sham acupuncture reduce muscle pain in myofascial pain patients J Orofac Pain 2002 16 71 76 11889662

[B35] Zhang YH Liu BY He LY Zi MJ Patient-reported outcomes: advances in research and practical application Zhong Xi Yi Jie He Xue Bao 2008 6 1101 1104 18990333 10.3736/jcim20091101

